# MATB for assessing different mental workload levels

**DOI:** 10.3389/fphys.2024.1408242

**Published:** 2024-07-23

**Authors:** Anaïs Pontiggia, Danielle Gomez-Merino, Michael Quiquempoix, Vincent Beauchamps, Alexis Boffet, Pierre Fabries, Mounir Chennaoui, Fabien Sauvet

**Affiliations:** ^1^ Département Recherche, Expertise et formation aéromédicale (REF Aéro), Institut de Recherche Biomédicale des Armées (IRBA), Brétigny sur Orge, France; ^2^ URP 7330 VIFASOM, Université Paris Cité, Hôtel Dieu, Paris, France; ^3^ Laboratoire IMS, CNRS, UMR 5218, Université de Bordeaux, Talence, France; ^4^ Thales AVS, Mérignac, France

**Keywords:** mental workload, MATB, monitoring, performance, NASA-TLX, pilot, electrophysiology, environmental constraints

## Abstract

Multi-Attribute Task Battery (MATB) is a computerized flight simulator for aviation-related tasks, suitable for non-pilots and available in many versions, including open source. MATB requires the individual or simultaneous execution of 4 sub-tasks: system monitoring (SYSMON), tracking (TRACK), communications (COMM), and resource management (RESMAN). Fully customizable, the design of test duration, number of sub-tasks used, event rates, response times and overlap, create different levels of mental load. MATB can be combined with an additional auditory attention (Oddball) task, or with physiological constraints (i.e., sleep loss, exercise, hypoxia). We aimed to assess the main characteristics of MATB design for assessing the response to different workload levels. We identified and reviewed 19 articles for which the effects of low and high workload were analyzed. Although MATB has shown promise in detecting performance degradation due to increase workload, studies have yielded conflicting or unclear results regarding MATB configurations. Increased event rates, number of sub-tasks (multitasking), and overlap are associated with increased perceived workload score (ex. NASA-TLX), decreased performance (especially tracking), and neurophysiological responses, while no effect of time-on-task is observed. The median duration used for the test is 20 min (range 12–60) with a level duration of 10 min (range 4–15). To assess mental workload, the median number of stimuli is respectively 3 events/min (range 0.6–17.2) for low, and 23.5 events/min (range 9–65) for high workload level. In this review, we give some recommendations for standardization of MATB design, configuration, description and training, in order to improve reproducibility and comparison between studies, a challenge for the future researches, as human-machine interaction and digital influx increase for pilots. We also open the discussion on the possible use of MATB in the context of aeronautical/operational constraints in order to assess the effects combined with changes in mental workload levels**.** Thus, with appropriate levels of difficulty, MATB can be used as a suitable simulation tool to study the effects of changes on the mental workload of aircraft pilots, during different operational and physiological constraints.

## 1 Introduction

The Multi-Attribute Task Battery (MATB) is a computerized flight simulator designed to evaluate operator performance and workload (Comstock and Arnegard, 1992). MATB provides a set of reference tasks analogous to the activities that aircrew members perform in flight, with freedom of use for non-pilot subjects. Simultaneous execution of multiple tasks is a central feature of MATB that corresponds to most operational systems and therefore makes the task useful for military purposes as a research tool for workload assessment. Mental workload can be characterized as the interaction between machine and task components, on the one hand, and the operator’s resource capabilities, motivation and state of mind, on the other (Hancock and Caird, 1993). A more precise definition of workload has been defined as the “costs” that a human operator incurs to complete an assigned task (Hart and Staveland, 1988; Longo et al., 2022). High workload levels could lead to errors and accident, while low workload could lead to frustration and lack of attention (Hancock et al., 1995). High and low mental workload are considered as a potential trigger for reduced pilot performance and higher accident risk (Morris and Leung, 2006). Automation, if properly designed, can reduce the human operator’s workload to a manageable level under peak load conditions.

Nevertheless, although the MATB has shown promise in detecting performance degradation due to high workload, fatigue, prolonged wakefulness, or physiological constraints during complex tasks, several studies have produced conflicting results or unclear regarding the number and type of MATB tasks to be used for assessment (Gutzwiller et al., 2014; Kong et al., 2022).

In this review, we aim to describe the methods used to assess physiological responses to increased multitasking workload using the MATB in order to provide recommendations for standardization of procedures, task duration, workload levels and analysis of results.

## 2 Review method

In a first part, we lead a systematic review based on the Preferred Reporting Items for Systematic Reviews and Meta-Analyses (PRISMA) standard (Page et al., 2021). A literature search was performed using PubMed, Google scholar, Scopus, ScienceDirect, IEEE and ArXiv, covering the period from 1 January 1990, to 31 December 2023. Only articles in English containing the terms “MATB”, “MATB-II”, “Multi-Attribute Task Battery”, or “Multi Attribute Task Battery” in their titles, abstract, keywords and text were considered during identification. The full text of the identified articles was then assessed for eligibility by two reviewers. Inclusion criteria for this review were workload-related full test articles, technical reports or short congress communications, using MATB, with at least two levels of workload (high and low). We excluded articles with only one workload level, with two operators, without methodological description and review articles. Selected articles were described in a table including training duration, task duration, event rate for each sub-task, analyzed performance, subjective scales, and recorded electrophysiological parameters. The recorded parameters were checked by another reviewer. We calculated for each MATB sub-task and for low and high mental workload levels, the mean, the median, the minimum and maximum event rate values observed in the literature. Correlations were made between event rates and MATB performance and NASA-TLX score.

In a second part, we aim to extend the literature review based on additional resources not selected by the procedure but nonetheless relevant to the subject, namely the interaction of MATB difficulty and some operational constraints such as hypoxia/altitude, diving, exercise, sleep deprivation. From the precedent literature search, we extracted articles containing MATB and physiological constraints. These articles have been excluded from the systematic review because different levels of MATB have not been used.

## 3 Systematic review results

### 3.1 Selected publications

**FIGURE 1:**
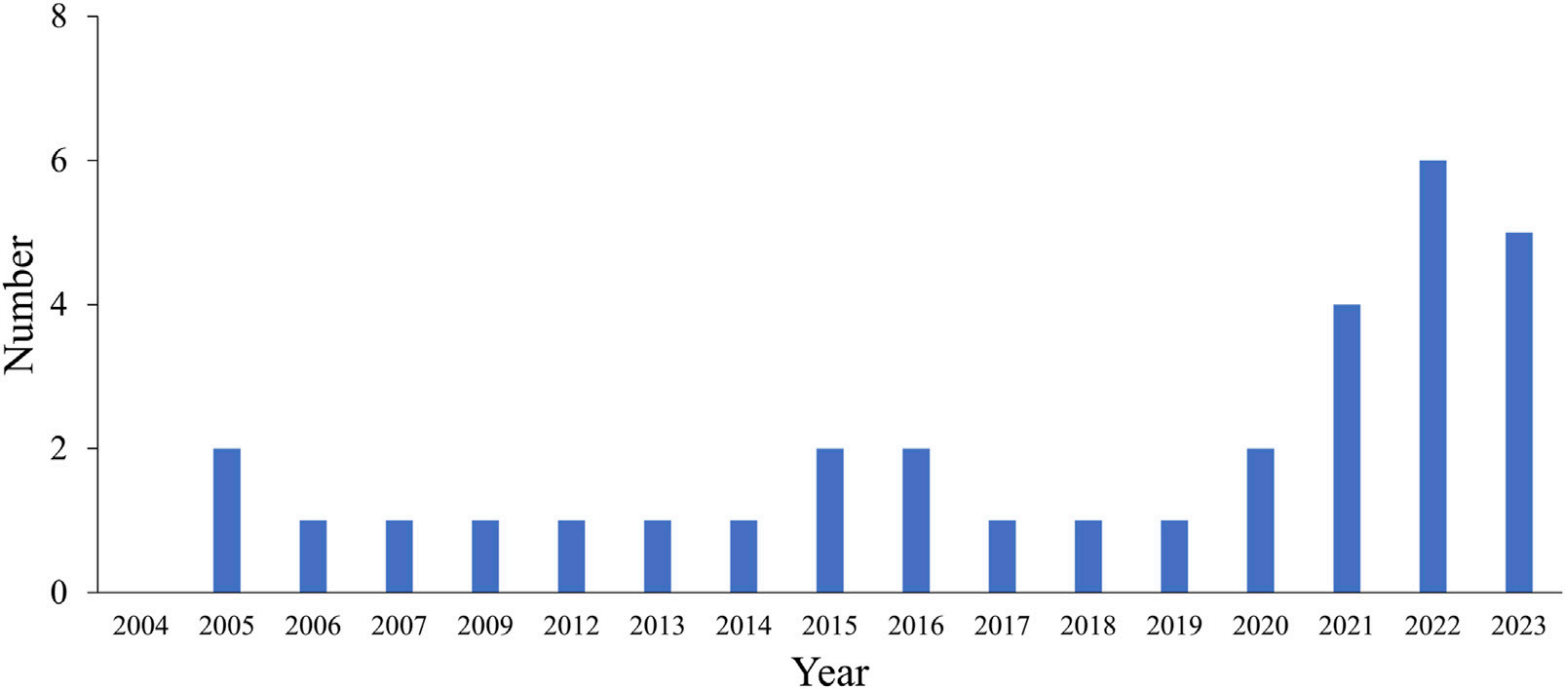
Number of MATB-related publications indexed in PubMed per year.

**FIGURE 2:**
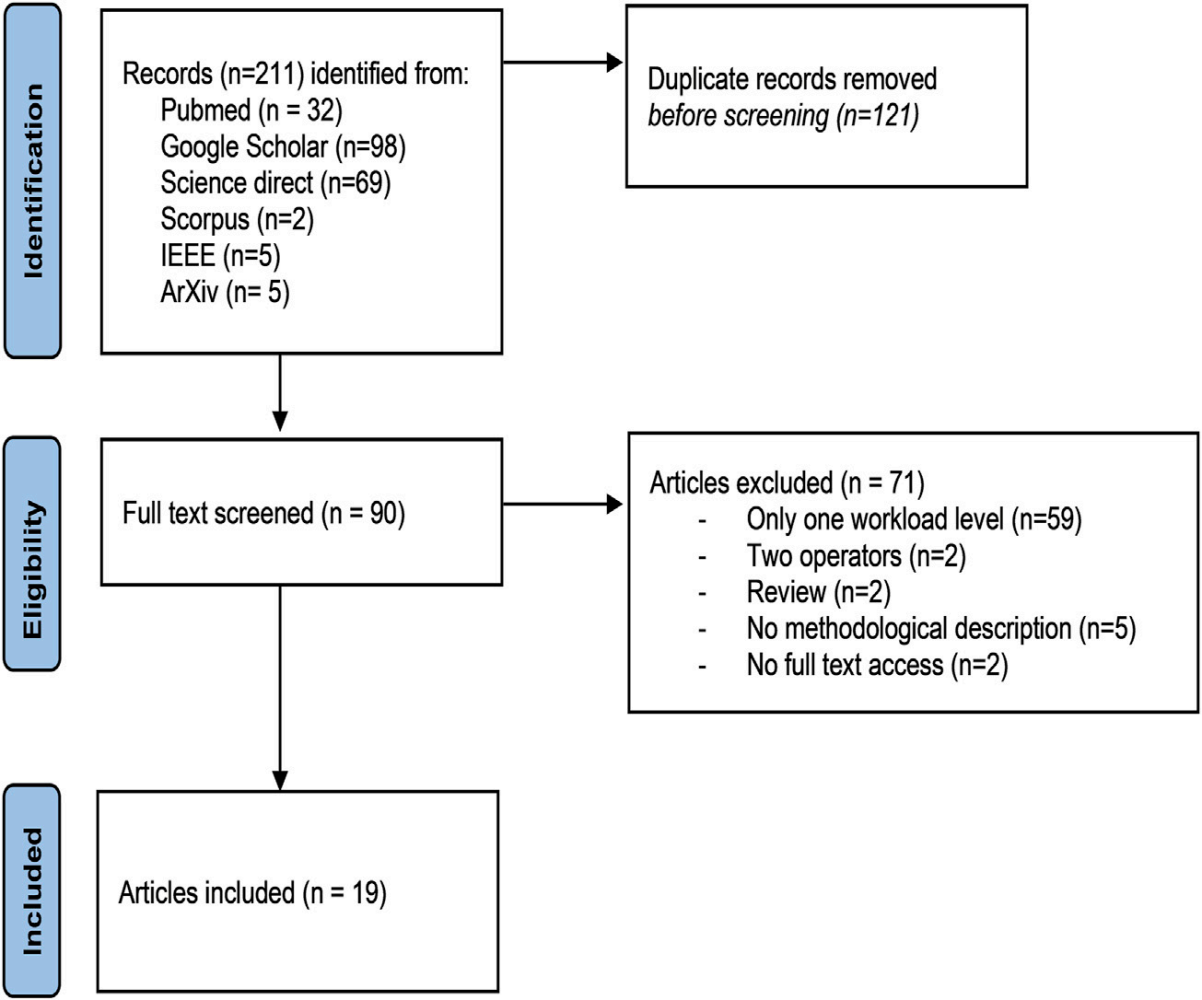
PRISMA flowchart illustrating the structured narrative review selection process.

The number of MATB-related publications has increased since 1992, as shown by PubMed (*n* = 32, since 2005) since 2005 (Figure 1), and particularly over the last decade. At the end of the selection process, only 19 publications with assessments of responses to increased mental workload (high vs. low) using the MATB were included in the tables. The flowchart for article selection is shown in Figure 2. Due to the small number of articles and the divergence of collected parameters, it was not possible to carry out a meta-analysis.

### 3.2 Description of MATB sub-tasks and outcomes

**FIGURE 3:**
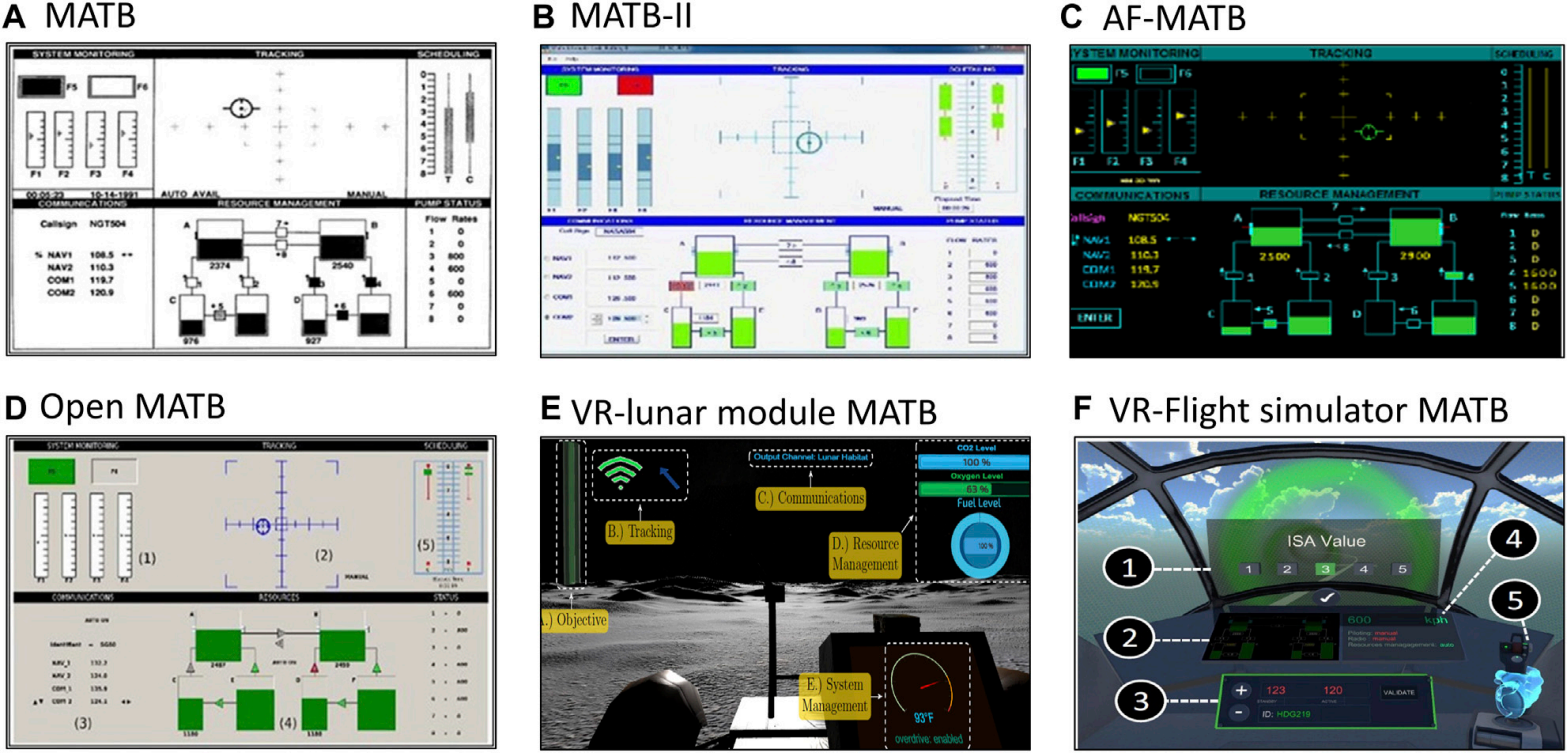
Screenshots of different versions of the Multi-Attribute Task Battery (MATB). **(A)** Original MATB (Comstock Jr and Arnegard, 1992), **(B)** MATB-II (Santiago-Espada et al., 2011), **(C)** Air Force (AF)-MATB (Miller Jr et al., 2014), **(D)** Open MATB (Cegarra et al., 2020), **(E)** Virtual Reality (VR)-lunar module MATB (Wilson et al., 2021), and **(F)** Virtual Reality (VR)-Flight simulator MATB (Luong et al., 2020).

The Multi-Attribute Task Battery was originally developed in the early 1990s by the Langley Research Center (LaRC) of the National Aeronautics and Space Administration (NASA) (Comstock Jr and Arnegard, 1992) and re-implemented under Microsoft Visual Studio.NET (VS.NET). The US Air Force has developed its own colorized version of MATB (AF-MATB) (Miller Jr et al., 2014), using colors similar to those of cockpit instruments for laboratory research. In 2011, the MATB (Santiago-Espada et al., 2011) was customized in a new colorized version (MATB-II), accessible *via* a website (https://matb.larc.nasa.gov/) and coded in C. The MATB-II runs on Windows-based computers with modern operating systems (compatible with the 64-bit version of Windows 7). Screenshots are shown in Figure 3. In 2020, Cegarra et al. proposed an open version (Open MATB), programmed in Python, and covered by a free software license to improve the use and replicability of the test (Cegarra et al., 2020). Recently, new versions of MATB have been developed using a virtual reality environment, but these versions are not available as open source (Luong et al., 2020; Wilson et al., 2021; Gozzi et al., 2022).

**FIGURE 4:**
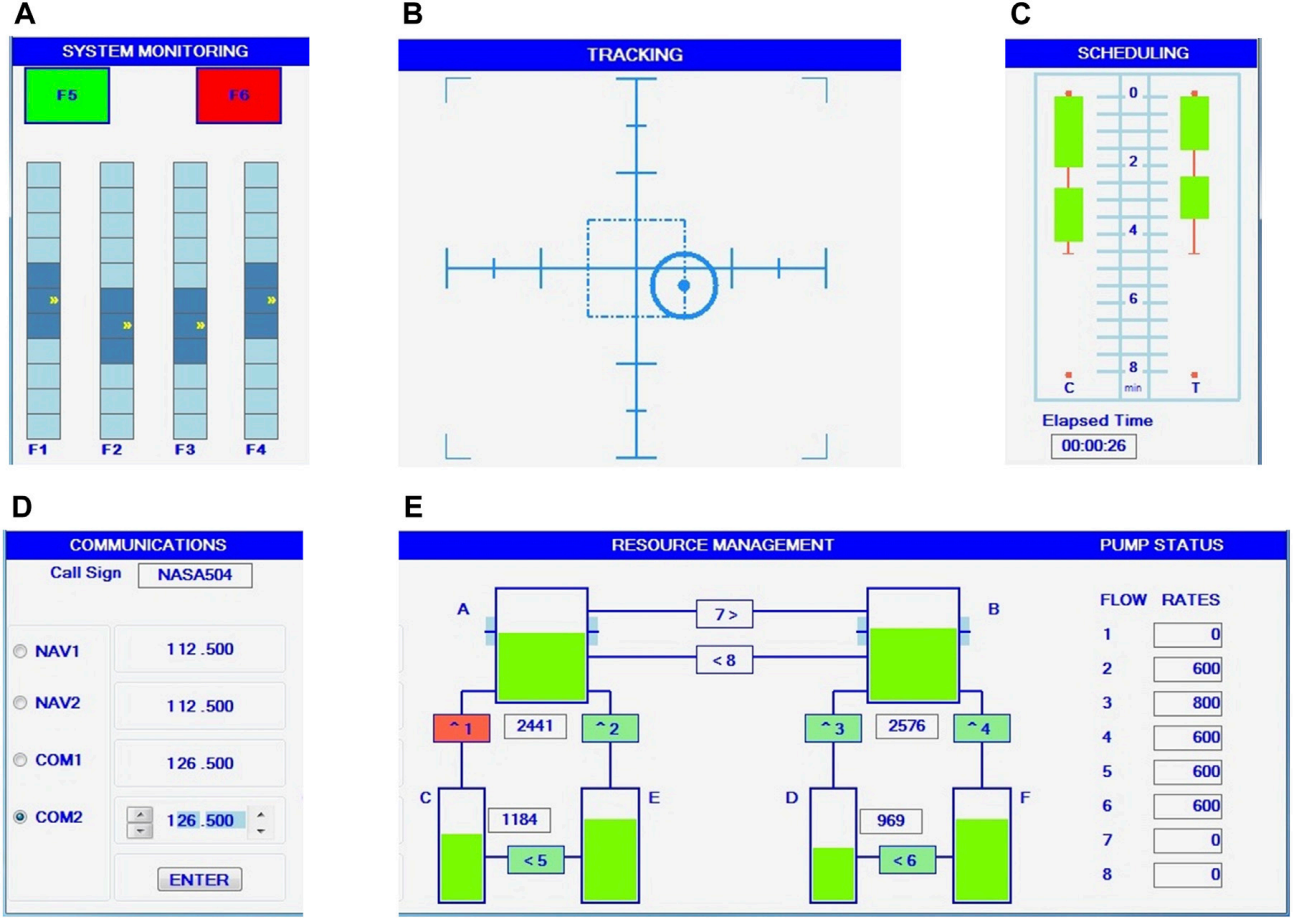
Illustration of the four sub-tasks of the Multi-Attribute Task Battery-II (MATB-II): MONITORING **(A)** task in the upper left corner where participants must respond as quickly as possible to lights and scale fluctuations *via* keystrokes, TRACKING **(B)** task in the upper middle window where participants must keep a circle as close as possible to the center with a joystick, COMMUNICATIONS **(D)** task in the lower left corner where participants should only respond to broadcast messages matching their call name, and RESOURCE MANAGEMENT **(E)** task in the lower right corner that requires participants to keep tank levels as close as possible to 2,500 by managing eight pumps. **(C)** is the workload rating survey (WRS), which automatically evaluates the temporal progress of the task. No action is required.

The MATB requires the individual or simultaneous performance of four sub-tasks (Figure 4): system monitoring (SYSMON), tracking (TRACK), communications (COMM), and resource management (RESMAN). A workload rating survey (WRS) provides feedback related to task progress. The investigator can determine test duration, number of sub-tasks used, event rates, response times and overlap, i.e. the possibility of observing a new stimulus before responding to the previous one (Santiago-Espada et al., 2011). The principle of multitasking is itself multifaceted, with sometimes supporting concurrent task performance, but often forcing sequential task operations. The first case has been well modeled by multiple resource theory (Wickens, 2008) and threaded cognition (Salvucci and Taatgen, 2011). The Multiple resource theory asserts that people have a limited set of resources available for mental processes, in particular during high mental workload. This theory explains how difficult single-tasks can run into processing difficulties and how dual-task performance is more likely to be hampered by performing similar tasks rather than dissimilar tasks (Wickens, 2002; 2008).

In the Open MATB version, investigators can easily define the MATB design by modifying a script file, without any action on the source code (Cegarra et al., 2020). The subject is instructed to perform tracking task and manage the other tasks. He/her has to carry-out all the subtasks, without prioritization instructions (Lewis et al., 2024). There is no indication of the analyzed result nor performance feedback (Santiago-Espada et al., 2011). The only feedback given to the subject is the temporal progression of the task.

#### 3.2.1 System monitoring (SYSMON, SYS or MON)

The system monitoring task (SYSMON task, abbreviated as SYSMON, SYS or MON in the literature), presented in the upper left window of the screen, tests attention and impulsivity by asking the subject to respond to the states of RED and GREEN “warning” lights, as well as to a group of four continuously moving scales. SYSMON lights and scale responses are combined to facilitate interpretation. The SYSMON task was rated using a methodology derived from the T.O.V.A.^®^ (Test of Variables of Attention, Clinical Manual) and adapted to MATB-II. The T.O.V.A.^®^ is a well-validated continuous performance test used for the diagnosis of attention deficits, including Attention-deficit/hyperactivity disorder (ADHD) (Greenberg and Waldmant, 1993). It compares a subject’s errors of omission and commission to normative data. The T.O.V.A.^®^ defines errors of omission as measures of attention or distraction and errors of commission as measures of impulsivity or disinhibition. Extremely frequent commission errors indicate a non-compliant gaming strategy in which the subject responds strategically by pressing several keys in an attempt to randomly obtain a greater number of correct responses. This strategy biases the overall score, as excessive commission errors reduce omission errors, shorten response times and increase variability (Grane et al., 2014).

The SYSMON outcomes are three performance indices: average reaction time (RT), accuracy (ACC) (i.e., the percentage of correct responses), and false alarm (FA) rate (i.e., the percentage of commission). These indices can be calculated separately for each button and scale, as well as for overall task performance (Table 1) (Gutzwiller et al., 2014; Chandra et al., 2015). In addition to these performance metrics, it would be interesting to analyze SDT (signal detection theory) metrics to separate discrimination certainty from response bias–factors that can independently affect accuracy. This theory proposes that one’s ability to detect a stimulus is not only based on the intensity of the stimulus itself but also on the psychological or physiological state of the person observing the stimulus (Green et al., 1966; Kim et al., 2014).

**TABLE 1:** MATB-II outcomes.

MATB-II task	Cognitive measurement	Outcome and formula
TRACKING (TRACK)	Visual perception Motor precision	Tracking score: root mean squared distance in pixels from the target center point to cursor (RMSE), calculated each 15 s
SYSMON (SYS or MON)	Inattention or distraction	Accuracy (ACC): (total number of events - number of missed events)/total number of events
SYSMON (SYS or MON)	Impatience or impulsivity	Commission errors, false alarms (FA): number of incorrect response/total number of events
SYSMON (SYS or MON)	Attention response speed	Reaction time: mean reaction time for correct responses
COMMUNICATION (COMM)	Auditory memory	Accuracy (ACC): number of correct responses to target message/total number of target messages
COMMUNICATION (COMM)	Auditory memory	Commission errors (false alarm) rate: number of responses to nontarget messages/total number of nontarget Messages
COMMUNICATION (COMM)	Auditory memory	Auditory reaction time: mean reaction time for correct responses
RESMAN	Strategy and planning	Deviation from target fuel level: root mean square error (RMSE) from the target fuel level, calculated by 30 s interval

#### 3.2.2 Tracking (TRACK)

The request for manual control is simulated by the tracking task, located in the upper middle window. Using a joystick, the subject keeps the target at the center of the window. The output parameter (tracking score) is the root mean square distance, from the center of the target to the cursor location (named Root Mean Square Deviation RMSD, or Error RMSE), calculated every 15 s by default (Santiago-Espada et al., 2011). This task is often the only constant task across different workload levels and could be used to assess the subject’s objective performance decrement. Tracking can be automated to simulate the reduced manual demands of autopilot and reduce mental load (Cegarra et al., 2020).

#### 3.2.3 Communications (COMM)

In the communications task (COMM), random pre-recorded voice messages announce call signs, as well as one of four possible radio channels and their frequencies (three integers and three decimal numbers). The messages are presented through the speakers of the experimental computer. The subject’s task is to determine which messages are relevant and respond to them by selecting the appropriate radio channel and frequency in the communication window. Other irrelevant calls are announced throughout the task (subjects must only respond to messages containing their call name, i.e., target messages). Responses are considered correct if the radio channel and frequency match those of the target message. The COMM task tests auditory discrimination and memory skills using a radio communications scenario in an aircraft control tower. The audio files used in the COMM sub-task can be customized to accommodate native language audio (Cegarra et al., 2020). The outcomes include the average reaction time (RT) and the calculated rate of correct and false responses. As for the SYSMON task, it would be interesting to investigate metrics of SDT.

#### 3.2.4 Resource management (RESMAN)

Fuel management requirements are simulated by the resource management task. The goal is to maintain the fuel levels in tanks A and B at 2,500 units each, which is achieved by controlling the operation of all eight pumps (on or off). Pump failures can occur and are indicated by red on the failed pump. The resource management window shows a diagram of the fuel management system. The six large rectangular regions are tanks containing fuel, and the green levels within the tanks represent the amount of fuel in each tank. The RESMAN task tests strategy and planning using an aviation fuel management scenario with failing fuel pumps. The outcomes are the normalized mean absolute value of deviation from the target fuel level for the A and B fuel tanks. According to Cegarra et al. (2020), performance may be considered on the top two tanks as the RMSE over a given period, by 30 s in the default configuration (Santiago-Espada et al., 2011).

#### 3.2.5 Additional tasks

In the study of mental workload responses, the MATB-II was used in combination with other supplementary tasks such as the auditory OddBall, which required no action on the part of participants, with to a constant event rate across different levels of workload. This combination enables mental workload to be assessed through electroencephalography (EEG) and auditory event-related potentials (ERPs) or task-irrelevant auditory ERPs (tir-aERPs) (Roy et al., 2016a; Xu et al., 2020; Ke et al., 2021). These features have shown great potential for building adaptive assistive human-machine systems by estimating mental workload in real time. However, extracting EEG features that consistently indicate mental workload across different tasks remains one of the key challenges. The use of these objectively validated estimators of mental workload increases the validity of MATB (Ke et al., 2021), and opens up the possibility of studies with simulated or real operational scenarios of increased mental workload, as done by Benthem et al. (2019).

### 3.3 Configuration of MATB for evaluating mental workloads

In publications, several factors are used to design different workload levels, including the overall duration of the task, the duration of individual levels, and the complexity of the task through adjustments in event rates and the number of simultaneous tasks (Table 2). We observed a significant variability between studies regarding the configuration of MATB. In 5 of the 19 studies, the MATB configuration was not described (Table 2). Only in 7 publications was it sufficiently detailed for potential replication or review.

**TABLE 2:** MATB configurations for mental workload evaluation.

Authors	Number of subjects	MATB version + Supp tasks	Training	Global test duration (min)	Level duration (min)	Task demand (mental workload) (Event rate per minute)				Levels order
							Low	Moderate	High	
Hancock et al. (1995)	15	**MATB** TRACK SYSMON RESMAN	Yes Duration not indicated	36	12		9 conditions (baseline association)			Random
						TRACK	3	6	8	
						SYSMON	0.5	1	1.5	
						RESMAN	0 .25	0.4	0.6	
Miyake et al. (2009)	15	**MATB** TRACK SYSMON RESMAN	3 sessions of 5 min	15	5	TRACK	1	2	4	High to Low
						SYSMON	Equal among the levels			
						RESMAN	Equal among the levels			
Bowers et al. (2014)	16	**AF MATB** TRACK SYSMON RESMAN COMM	6 sessions of 6 min (in 2 h)	24	6		Not indicated		individualized	Random
Chandra et al. (2015)	10	**MATB-II** TRACK SYSMON RESMAN COMM	Yes Duration not indicated	16	8		Not indicated		Not indicated	Low to High
Roy et al. (2016a)	8	**MATB-II** TRACK SYSMON RESMAN + Oddball	Not indicated	20	10	MATB-II Oddball	2 tasks SYSMON RESMAN >3		3 tasks TRACK RESMAN SYSMON >3	Random
Charles and Nixon (2017)	44	**MATB-II** TRACK SYSMON RESMAN COMM	Not indicated	30	5		Not indicated		Not indicated	Random
Kim et al. (2019)	20	**AF-MATB** TRACK SYSMON RESMAN COMM	≈15 min	12	4	SYSMON RESMAN COMM	≈13.7 ≈2.0 ≈1.5		≈35.7 ≈7.3 ≈4.25	Low-High-Low
Mortazavi et al. (2019)	13	**MATB-II** TRACK SYSMON RESMAN COMM	Yes Duration not indicated	15	5	Global TRACK	0.5 bit/s Automatic	1 bit/s Manual	1.5 bit/s Manual	Not indicated
Benthem et al. (2019)	51	**MATB-II** TRACK SYSMON RESMAN COMM	Not indicated	Not indicated	Not indicated		Not indicated	Not indicated	Not indicated	Not indicated
Zhang et al. (2020)	15	**MATB-II** TRACK SYSMON RESMAN COMM	Yes Duration not indicated	36	12	SYSMON RESMAN COMM	0.2 0.2 0.2	1 1 1	3 3 3	Random
Xu et al. (2020)	10	**MATB-II** TRACK SYSMON RESMAN + Oddball	individualized	20	10	TRACK	Low		High	Random
						SYSMON	2		20	
						RESMAN Oddball	1 5.2		3.5 5.2	
Qu et al. (2021)	15	**MATB-II** TRACK, SYSMON RESMAN	Not indicated	24	12	TRACK SYSMON RESMAN COMM	Automatic 1 1 1		Manual 24 24 24	Random
Huang et al. (2021)	15	**AF-MATB** TRACK SYSMON RESMAN COMM	Yes Duration not indicated	20	10	TRACK SYSMON RESMAN COMM	Easy 8 2 2.5		Hard 40 16 9	Random
Ke et al. (2021)	17	**MATB-II** TRACK SYSMON RESMAN + Oddball	Yes Duration not indicated	40	10	MATB-II TRACK SYSMON RESMAN Oddball	2 tasks Easy 2 1 2–3		3 tasks Hard 20 3.5 2–3	Random
Rosanne et al. (2021)	48	**MATB-II** TRACK, SYSMON RESMAN + physical activity	Yes Duration not indicated	60	10		Not indicated		Not indicated	Not indicated
Kong et al. (2022)	20	**MATB-II** SYSMON RESMAN COMM + sleep deprivation	40 min	45	10 (low) 15 (high)	SYSMON RESMAN COMM	1 task 6 8 2		3 tasks 6 6 2	Low to High
Lam et al. (2022)	24	**MATB-II** SYSMON RESMAN COMM	Not indicated	20	5	SYSMON COMM	6 1.2		12 3	Random
Zhang et al. (2023)	10	**MATB-II** TRACK, SYSMON RESMAN	Not indicated	Not indicated	Not indicated	Global	1		24	Not indicated
Chen et al. (2023)	17	**MATB-II** TRACK SYSMON RESMAN	Yes Duration not indicated	20	10	TRACK SYSMON RESMAN	Automatic 2 1		Manual 20 3.5	Random

#### 3.3.1 Level duration

The median test duration observed is 20 min (range: 12–60) with a median level duration of 10 min (range: 4–15) (Table 3). In many studies, the duration of each level was equal in low and high mental workload tasks. The impact of task duration is an interesting but complex subject in aeronautical simulation. An increase in mental workload has been observed for levels of less than 5 min (Kim et al., 2019), as well as for tasks longer than 12 min (Kong et al., 2022). Bowers et al. (2014) observed an increase in false alarms during the first 3 min of a high workload period, and stability was observed after 4 min of testing (Bowers et al., 2014). Moreover, it has been observed that switch resistance during tasks show no decrease over time overall, and there was no effect of time on task during MATB (Fairclough et al., 2006; Gutzwiller et al., 2014; 2016). Nevertheless, a minimum duration of 10 min is required to observe at least two target stimuli in the communication task, with a minimum of two events per minute and 25% of targets, which is a common configuration for low-level mental workload (Kong et al., 2022). In the particular context of the sleep loss protocol, it was observed that durations of less than 10 min underestimated the impact of sleep loss on performance. This was particularly evident in measures of reaction time and tracking deviations (Caldwell et al., 2004).

**TABLE 3:** Average MATB configurations for mental workload evaluation in the literature.

	Subject number	Training duration (min)	MATB duration (min)	Level duration (min)	Task demand (mental workload) (Event rate per minute)	
					Low	High
Mean	20.2	26.5	26.6	8.8	6.3	25.4
Median	15	25.5	20	10	3	23.5
Minimal	8	15	12	4	0.6	9
Maximal	51	40	60	15	17.2	65

#### 3.3.2 Number of tasks

According to a standard definition, multitasking is a situation in which people manage several distinct tasks in a given time interval, and have to switch from one task to another according to available priorities (Oswald et al., 2007). In fact, it has been proven that the divided attention that arises in multitasking supervision of a system imposes a greater workload on humans than the sustained attention required for a single task (McDowd, 2007). In MATB, the difference between the low and high workload conditions could be imposed by additional auditory stimuli (Roy et al., 2016a) or an increased event rate in a fixed number of tasks (Kim et al., 2019). In workload studies, the average number of tasks used is between 3 and 4.

#### 3.3.3 Event rates

Increasing the event rate is probably one of the most widely used methods to increase the mental workload induced by MATB (Table 2). The difficulty of each task was determined by adjusting the event rate in many studies. The median duration used for the test is 20 min (range 12–60) with a level duration of 10 min (range 4–15). To assess mental workload, the median number of stimuli is respectively 3 events/min (range 0.6–17.2) for low, and 23.5 events/min (range 9–65) for high workload level. The total event rate is calculated by summing all sub-task events per minute. In this review, the lower value of events rate has been observed when MATB is associated with high levels of carbon dioxide (Zhang et al., 2020). In other studies, lower values have been used when MATB is associated with environmental/operational constraints such as sleep deprivation (Caldwell et al., 2004), hypoxia (Van Dorp et al., 2007; Bottenheft et al., 2023). We observed (Table 2) that for 6 of the 19 studies, it was not possible to determine the total event rate. For 9 of the 19 studies, it was not possible to assess the exact event rate for each MATB sub-task. In one study, the event rate was customized and calculated based on the performance achieved during training (prior to the testing session) (Bowers et al., 2014). Knowledge of event rates is necessary for MATB design. A more precise systematic description of the event rate for each sub-task, including the percentage of targets and non-targets, is needed for the reproducibility of publications.

#### 3.3.4 Transitions

Bowers et al. (2014) studied the effects of workload transitions (i.e. easy-to-hard vs. hard-to-easy) on participants’ performance and neurophysiological signals, using the Tracking and SYSMON tasks in the AF-MATB. With regard to performance, they showed that most of the significant differences between conditions were between the easy and hard portions of the trials. However, they also showed that performance in the hard-to-easy transitions in the two tasks was significantly worse compared to the consistently easy trials (Bowers et al., 2014). The EEG frontal (Fz) theta activity was overall higher in the hard trials compared to the easy ones, with rapid and complete crossover after transition (easy-to-hard and hard-to-easy). The lateral (T5) gamma activity exhibited a strong decline at the beginning of the run that stabilizes at a higher power in the hard than in the easy trial, and the transition conditions produced a crossover, but the change following a hard-to-easy transition was relatively slow. The authors concluded that the EEG analysis provides some additional support for slow adaptation to a sudden decline in workload, but still pointed out that previous research has been rather mixed as to which types of transitions have an impact on performance (Bowers et al., 2014). In this study, the analysis of the NASA-TLX index and the shortened version of the Dundee Stress-State Questionnaire (Matthews et al., 1999) did not reveal any significant differences related to workload transitions. Thus, the results and analysis of the study by Bowers et al., 2014 prompt us to suggest that a randomized design between low and high mental workload may be a strategy to reduce possible transition-induced biases.

#### 3.3.5 Overlap

MATB can be designed with one or more stimuli simultaneously (called overlap), or with a refractory inter-stimulus period without overload. Overlap stimuli increase difficulty and mental workload (Gutzwiller et al., 2014; Lam et al., 2022) and are mainly observed at higher levels of mental workload (Lam et al., 2022).

#### 3.3.6 Feedback

The impact of two approaches, feedback (in the form of success or failure in task performance) and reward or punishment (in monetary form), can affect performance control (Neal and Griffin, 2006; van den Berg et al., 2014), however few studies have evaluated this in the context of MATB. Some studies observed that performance feedback had no significant effect on mental workload and malfunctions detection (Singh et al., 2005; 2010). However, Calabrese et al. (2023) compared four different randomized groups (no intervention, punishment, feedback, punishment + feedback) during MATB-II. They showed that punishment, feedback, and punishment + feedback decreased errors and increased performance, with punishment alone having the greatest effect (Calabrese et al., 2023). These results underline the value of behavioral consequences of feedback in reducing errors. Financial reward, proportional to the rate of correct response, is thus a habitual procedure in studies of mental fatigue to improve motivation (Smith et al., 2019), although the tasks are longer and often more boring than the MATB task. Another recent study evidenced the beneficial effect of a gamification method (i.e. feedback score, with points allocations, a key factor in the gamification method, and leaderboard as an additional motivating factor) on the performance of every single task of the MATB (Stasch and Mack, 2023). Thus, in all studies using feedback, punishment or financial reward, the methodology must be clearly described.

#### 3.3.7 Training

In 13 of the 19 studies, a training session was included in the protocol with a minimum duration of 15 min, more than 30 min for 2 of them, and no fixed duration (*ad libitum*) in 2 studies. No training session was described in 6 studies. In the study of Fairclough dans Venables (2006), during the training period, naive participants were instructed about the objectives of each experimental task and then performed a 5-min familiarization session followed by 20 min of high-level training. In the study of Miyake et al. (2009), the subjects had a training session consisting of a three 5-min trials at a medium level before the experiment, and they were given guidance on the task so that they could learn it easily (Miyake et al., 2009). Freiberger et al. (2016) observed that at least two short (5 minutes) sessions of training were needed to observe good tracking performance. In the study of Kim et al. (2019), following the pre-experimentation survey, participants proceeded to a training session to familiarize themselves with the AF-MATB experiment tasks. Training was repeated until participants reached an average level of around 65% correct responses in the SYSMON and COMM tasks, and this was achieved over an average training session lasting 15 ± 3 min (6 ± 2 trials) (Kim et al., 2019). Singh et al. (2005) examined the effect of training on workload in flight simulation task (a revised 1992 MATB version) performance before and after a 30-min (short) and a 60-min (long) session of manual training. Mean detection performance showed marginally higher task performance in the long compared to the short training session, but the difference between the two training conditions was not significant. Authors suggested that the amount of training did not affect subjects’ SYSMON task performance. However, results also indicated that subjects reported significantly higher subjective temporal workload between pre- and post-test sessions in the short compared to the long training condition, and further subjects showed a significantly high degree of frustration workload in pre-than post-automated task performance in the long training condition (Singh et al., 2005). In conclusion, the optimal duration of training is not clearly defined in the literature. When designing a MATB script file, we recommend systematically carrying out a training session at different workload levels. This training protocol must be clearly described.

### 3.4 Prediction of mental workload levels

The definition of mental workload includes both behavioral and subjective aspects (Kramer et al., 1995). Optimal mental workload is a relevant topic for all kinds of work, but especially for jobs with high responsibilities like pilots. As a result, psychophysiological markers that reliably characterize mental workload levels are very useful, such as cardiac and vascular variables, electroencephalogram (EEG) and eye movement variables, and those derived from questionnaires linked to the perceived severity of the task performed (such as NASA-TLX) (Miyake et al., 2009; Bowers et al., 2014). In order to address the challenge of validating increased mental workload, a combination of behavioral (performance), subjective and electrophysiological measures was carried out (Table 4).

**TABLE 4:** Effects of increased mental workload (high *versus* low) on performance, subjective workload and electrophysiological responses.

Authors	MATB outcomes			Subjective scales		Electrophysiological measurements		
	TRACK	SYSMON	RESMAN	NASA-TLX	Others	ECG	EEG	Others
Hancock et al. (1995)	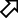 RMSE	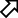 R.Time 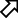 False alarm 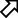 Omission	=	no	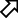 (SWAT)	no	no	no
Miyake et al. (2009)	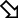 RMSE	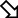 R.Time	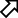 Accuracy	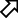	= (POMS, SACL, STAI-S)	yes	no	EDA, Breathing, BPV
Bowers et al. (2014)	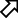 RMSE	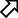 R.Time 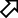 False alarm	Not indicated	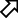	not indicated (DSSQ)	Yes (data not shown)	PSD: 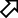 Theta (frontal) 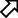 Gamma (temporal) = Alpha (parietal)	EOG (data not shown)
Chandra et al. (2015)	= RMSE	= R.Time = Accuracy	Not indicated	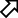	no	no	PSD: 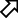 Beta energy (AF3 channel) 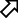 Beta/(alpha + Theta) (AF3, AF4, F7, F8)	no
Roy et al. (2016a)	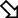 Global score			no	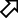 (Rating Scale Mental Effort)	no	ERP: 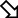 P200 latency Fisher LDA classification	no
Charles and Nixon (2017)	Not indicated	Not indicated	Not indicated	Yes (not indicated)	no	no	no	Eye-Tracking
Kim et al. (2019)	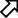 RMSE	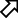 R.Time	Not indicated	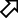	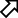 (ISA rating)	no	PSD: Adaptive Control of Thought- rational (ACT-R) model	no
Mortazavi et al. (2019)	Not indicated	Not indicated	Not indicated	no	no	no	no	no
Benthem et al. (2019)	Not indicated	Not indicated	Not indicated	Yes (not indicated)	no	yes	yes	no
Zhang et al. (2020) Zhang (2021)	U-shape Global score			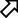	no	Yes	PSD: 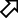 Beta relative power	Breathing
Xu et al. (2020)	Not indicated	Not indicated	Not indicated	no	no	no	Tir-aERPs: 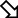 amplitude N100, RON, and amplitude P300	no
Qu et al. (2021)	Not indicated	Not indicated	Not indicated	no	no	Support vector machine modelling	no	no
Huang et al. (2021)	Not indicated	Not indicated	Not indicated	no	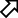 (Bedford scales)	no	PSD: Dynamic Causal Modeling	no
Ke et al. (2021)	Not indicated	Not indicated	Not indicated	no	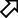 (Rating Scale Mental Effort)	no	PSD: 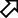 Theta (frontal, parietal), 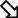 Alpha (frontal) Tir-aERP: 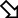 amplitude N100, P300, RON	no
Rosanne et al. (2021) Albuquerque et al. (2020)	Not indicated	Not indicated	Not indicated	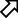	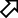 (Borg Scale)	Yes	PSD: Random forest and Support vector machine modelling	EDA, Breathing, Skin temperature, BVP
Kong et al. (2022)	no	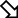 Accuracy	Not indicated	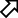	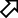 (Sleepiness score)	no	no	no
Lam et al. (2022)	= Global score accuracy			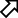	no	no	no	fMRI, Neural networks model
Zhang et al. (2023)	Not indicated	Not indicated	Not indicated	Yes (not indicated)	not indicated (SWAT and Workload Profile)	no	PSD: Support vector machine classification (EEG graph convolution neural network model)	no
Chen et al. (2023)	Not indicated	Not indicated	Not indicated	no	no	no	Topographies and dynamics of EEG microstates	no

Abbreviations: EDA, electrodermal; EOG, electrooculogram; R. Time, Response time; RMSE, root mean square error; SWAT, subjective workload assessment technique; SACL, stress arousal checklist; STAI-S, state and trait anxiety inventory; POMS, profile of mood states; DSSQ, dundee stress state questionnaire; ISA, Instantaneous self-assessment of workload; PSD, power spectral density; ERP, event related potential; tir-aERPs, task-irrelevant auditory ERPs; RON, the reorienting negativity; LDA, linear discriminant analysis; fMRI, functional magnetic resonance imaging; BPV, blood pressure variability.

#### 3.4.1 MATB performance measurements

**FIGURE 5:**
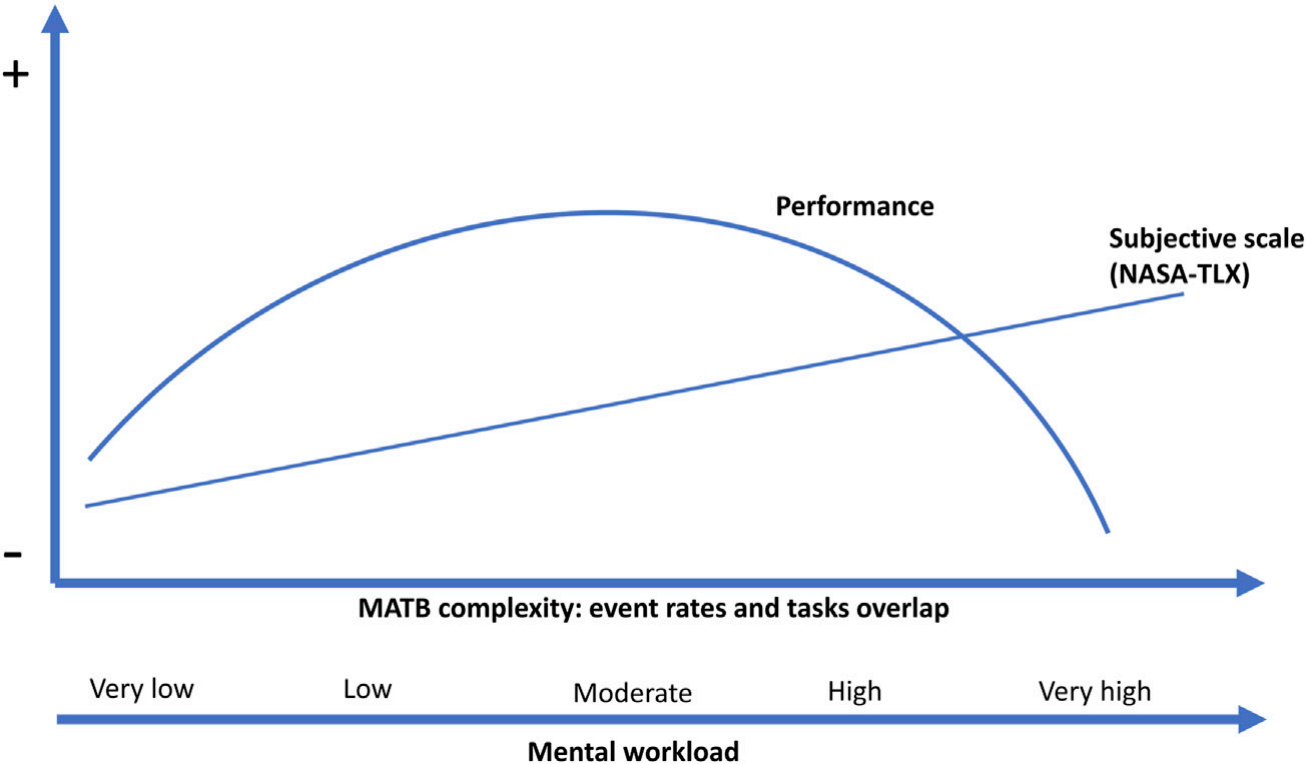
Performance and subjective scale evolution with increasing mental workload.

The mental workload of the task was recently shown to have a significant inverted U-shaped effect (Figure 5) on MATB task performance, and authors indicated that moderate mental workload was conducive to improved task performance, consistent with previous studies (Zhang et al., 2020). Both very low and high mental workload have been associated with decreased performance (Gutzwiller et al., 2014). The inverted U-shape effect was first described by the Yerkes-Dodson Law, showing the same effect between performance and arousal (Yerkes et al., 1908). A lower level of arousal observed on more difficult tasks than easier tasks (“task difficulty” hypothesis), brings a valuable framework to better understand the relationship between arousal and human performance. The hypothesis is supported by studies which have observed optimal performance at 60% and 70% of maximum arousal (Arent and Landers, 2003). In a recent review, Dehais et al. (2020) provided a framework to disentangle those neural mechanisms that underpin the relationship between task demand, arousal, mental workload and human performance, and advocated targeting those specific mental states that precede a reduction of performance efficacy. This can be approach through physiological and neurophysiological measures such as 1) heart rate (HR) and heart rate variability (HRV) for the activation or co-activation of the two branches of the autonomous nervous system (i.e., sympathetic or parasympathetic), 2) spectral analyses on the EEG signal, and also through behavioral metrics such as ocular behavior which can complement the detection of low and high levels of engagement, with eye tracking metrics.

In the Zhang et al. (2020) study, the difference between low and high mental workload was not significant. In their study, optimal performance was observed with a moderate event rate (Zhang et al., 2020). This methodological point is fundamental for the design of the MATB to observe a significant decrease in performance and a significant difference between the MATB levels. This could be achieved by using an additional level of moderate workload as in Zhang’s study (Zhang et al., 2020). Furthermore, evaluating different performance levels requires the use of a continuous task between levels, such as tracking or system monitoring. In the low mental load condition, the number of targets can be very low, as in communications and resource management tasks, leading to an increase in the weight of each error. Tracking with the same level of difficulty appears to be a an index of performance for workload assessment (Miyake et al., 2009; Bowers et al., 2014; Charles and Nixon, 2017; Kim et al., 2019). The workload effect can also be evaluated with the Oddball additional task if the same event rate is used (Roy et al., 2016; Xu et al., 2020; Ke et al., 2021). Considering all the publications listed in Table 2, the mean RMSE for the tracking task, observed in the literature is 32.1 (range: 10–46) for low workload and 56.3 (range: 45–87) for hard mental workload, with a mean difference of 23.1 (range: 28–46). We observed a significant positive linear correlation between tracking performance and the global event rate (*R* ^2^ = 0.52 *p* = 0.02 for low and *R* ^2^ = 0.71 *p* < 0.01 for high mental workload) considering both low and high mental conditions. Only four studies included a moderate mental workload level, but none of them produced results on RMSE.

#### 3.4.2 Subjective measurements

Many authors have tested their MATB configuration using the visual analogue scale (VAS), Bedford scales (Huang et al., 2021) or the NASA Task Load Index (NASA-TLX) that is the most frequently used. The NASA-TLX has been widely used to assess pilot task performance. Developed by the Human Performance Research Group at NASA’s Ames Research Center (Hart and Staveland, 1988), the NASA-TLX is a multi-dimensional rating scale. A weighted average of ratings on six subscales provides an overall workload rating. These subscales are: Mental Demand, Physical Demand, Temporal Demand, Own Performance, Effort, and Frustration. Subjects are required to rate their perceived effort on five of these subscales (except for their Own Performance) on a scale ranging from “low” to “high.” The Own Performance subscale ranges from “good’ to “poor.”. The NASA-TLX rating scale can be presented to the subject at any time during MATB battery operation. A code for the onset of rating scale presentation can be added to the script that generates events for the MATB Battery (Roy et al., 2016a; 2016b; Rosanne et al., 2021). In the 19 articles analyzed in Table 2, the mean NASA-TLX scores were 25.3 (range: 10–56) for the low mental load task and 62.8 (range: 45–77) for the hard task. The mean difference was 37 (range: 20–70). We observed a significant positive linear correlation between NASA-TLX and event rates for low mental workload (*R* ^2^ = 0.23, *p* = 0.04) and for high mental workload task (*R* ^2^ = 0.74, *p* < 0.01).

#### 3.4.3 Cardiac and vascular electrophysiology

It has been shown that cardiac assessment can be a useful complement to self-report measures for determining the mental workload associated with flying tasks, and the risk of performance decrements. However, regarding heart rate (HR), some studies show that it can distinguish different levels of mental workload in simulated flights, while others do not (Charles and Nixon, 2019; Li et al., 2022). In the 19 articles selected, the MATB-induced increase in mental workload was not associated with changes in HR or the coefficients of variation of R-R intervals (Miyake et al., 2009). However, in the spectral analysis of heart rate variability (HRV), an increase in indexes of sympathetic activity, such as low-frequency (LF) and the LF/HF ratio was observed along the MATB levels, as well as in the laser Doppler tissue blood flow and the skin potential level (SPL). Additionally, a significant test-retest correlation was obtained for the skin potential level (SPL) for more participants than for the other parameters, although there were large individual differences (Miyake et al., 2009). The HRV analysis is one of the most frequently employed physiological assessments of mental workload (Charles and Nixon, 2019). HRV indexes appear to be very sensitive to task-rest differences but less sensitive to increased difficulty levels within the same type of task (Jorna, 1992). Moreover, HRV is nonspecific to mental workload and affected by many mental states. Moreover, HRV spectral analysis needs at least 5 min of artifact-free recordings (Malik, 1996). In order to improve this, multiple features of ECG signals including R-R interval feature, ECG T and P wave power, QRS complex power and Sample Entropy (SampEn), have been associated with increased prediction of mental workload (Qu et al., 2021).

#### 3.4.4 Eye tracking

Eye tracking can be used to distinguish high and low workload levels. In particular, it has been shown that blink rate decreases during MATB (Fairclough and Venables, 2006), during a high-complexity task in a simulated nuclear control context (Hwang et al., 2008), and during a 90-min flight scenario (Wilson, 2002). During the MATB task, blinks count can differentiate between workloads (high or low) and task types, and reflect subjective workload ratings (Charles and Nixon, 2017). In particular, blinks count were significantly lower during tasks involving high visual load (TRACK, SYSMON and RESMAN) when compared to less visually demanding tasks (COMM); in addition, lower numbers of blinks were observed at higher workloads for all tasks with higher visual load (Charles and Nixon, 2017). The NASA-TLX scores were significantly negatively correlated with the mean number of blinks across all dimensions for the TRACK task and for the mental demand dimension for the SYSMON and RESMAN tasks (Charles and Nixon, 2017).

With regard to mental workload, ocular activity has been associated with different engagement-related mental states, such as mind wandering and effort withdrawal for disengagement, and perseveration, inattentional blindness and deafness for over-engagement (Dehais et al., 2019). In subsequent studies that did not use the MATB tool, the decrease of pupil diameter was shown related to mind wandering and inattentional deafness (Grandchamp et al., 2014), the increase of maximum pupil diameter with effort withdrawal (Peavler, 1974; Causse et al., 2016).

#### 3.4.5 Cerebral electrophysiology

Recent technological advances have made it possible to develop low-cost, highly portable brain sensors, such as pre-amplified dry-electrodes, for measuring cognitive activity outside the laboratory. These technologies opens up promising prospects for monitoring the “brain at work” in complex real-life situations, such as piloting an aircraft (Dehais et al., 2019). Mental load is a mental state that is currently one of the main areas of research in neurophysiology, thanks to electroencephalography (EEG) measurements, a method that enables the direct assessment of mental state. Estimators of increased mental workload are based on spontaneous EEG power spectral density (PSD), event-related potentials (ERPs) after auditory stimuli and task-irrelevant auditory ERPs (tir-aERPs) (Xu et al., 2020; Ke et al., 2021).

**FIGURE 6:**
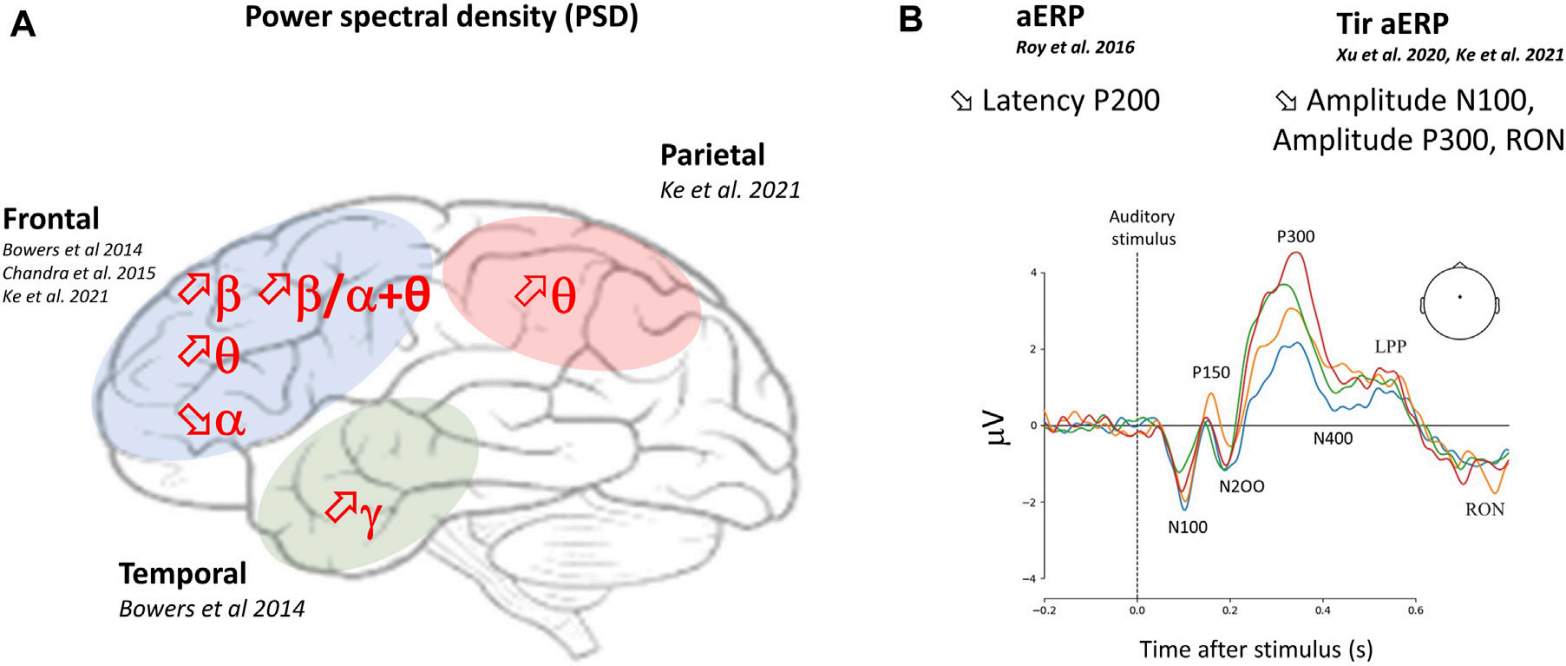
**(A)** Most affected brain areas by increased MATB mental workload and EEG power spectral density (PSD) responses. **(B)** Example of Cz EEG event-related potentials (ERPs) components during different levels of mental workload (from low in red, to high in blue). ERPs represent a measure of cortical electrical activity evoked during a cognitive task and recorded from the scalp with EEG. Each component is described by a letter indicating its polarity (P: positive and N: negative) and by a number/letter indicating its position in the sequence or a number indicating its latency. RON: the reorienting negativity, also called contingent negative variation (CNV), and LPP is the late positive potential. References related to Auditory (a)ERP and Task-irrelevant (Tir)-auditory (a)ERPs (Tir-aERPs) responses during increased mental workload are noticed.

During a real flight scenario, Dehais et al. (2019) evidenced the potential of dry-EEG electrodes to monitor cognitive activity in a highly ecological and noisy environment (Dehais et al., 2019). They showed higher P300 amplitude for the auditory target (Pz, P4 and Oz electrodes) along with higher alpha α) band power (Pz electrode), and higher theta θ) band power (Oz electrode) in the low load compared to the high load condition (Dehais et al., 2019). The relative PSD in theta, alpha, and low beta bands were also sensitive to MATB-induced increased mental workload in the laboratory (Figure 6A). The relative PSD in theta, alpha, and low beta bands were also sensitive to MATB-induced increased mental workload in laboratory studies. Higher overall frontal theta activity was observed in hard trials compared to easy trials of the MATB (Bowers et al., 2014; Ke et al., 2021).

Beta energy and the beta/alpha + theta ratio, also known as the activation index, increase in the frontal area (Chandra et al., 2015). At the same time, a decrease in alpha activity has been observed during the hard compared to low level of difficulty MATB in the frontal cortex (Ke et al., 2021). Manipulations of the difficulty of the AF-MATB task have been shown to be reflected in performance (correct RT and tracking RMSE) and mental workload (NASA-TLX) throughout the duration of an experimental condition, comparing human data with that of the high level of ACT-R (Adaptive Thought-Rational Control) known for its robustness in modeling cognitive processes (Kim et al., 2019). In a recent article, the temporal domain graph features of the EEG signal are used as the input features of a Graph Convolutional Network (GCN) model, and the high and low mental workload have been classified using EEG data collected by the MATB-II platform (Zhang et al., 2023). Dynamic causal modeling (DCM) for EEG has also shown interesting results in investigating the lateralization and direction of causal neural connections when mental workload is at different levels using the AF-MATB (Huang et al., 2021). Finally, using MATB, it was suggested that EEG microstates can provide valuable information about neural activity patterns with dynamic temporal structure at different levels of mental workload, and could be adapted for classification (Chen et al., 2023).

The above results are consistent with the scientific literature. Indeed, EEG power in the alpha band (8–13 Hz) has been found to be negatively correlated with mental workload in tasks such as working memory (Sauseng et al., 2005), simulated driving (Yang et al., 2018), and multitasking (Puma et al., 2018). EEG power features in frequencies ranging from 0.5 to over 100 Hz have been found to be modulated by task difficulty and used to estimate mental workload with machine learning techniques in many different tasks (Charles and Nixon, 2019; Chen et al., 2023). The satisfactory performance of EEG-based mental workload estimators trained and tested in the same task (within-task) shows their potential for practical application.

However, cross-task application, in which mental load estimators are trained on one set of tasks and applied to other tasks, remains a challenge. Perhaps the main reason for this challenge lies in the difference in neurophysiological responses between different types of tasks (Baldwin and Penaranda, 2012; Ke et al., 2014; 2015), linked to the different cognitive strategies used and associated with variations in EEG features. A significant main effect of task has been observed for spectral power, especially in the alpha band (Baldwin and Penaranda, 2012; Ke et al., 2014). The poor performances observed using the EEG spectrum model in measuring inter-task mental workload (Baldwin and Penaranda, 2012; Ke et al., 2014; 2015) limit its generalization.

The theory of cognitive resources and its relation to the generation of event-related potentials (ERPs) may provide new EEG features (Roy et al., 2016b; 2016a) that are more robust and sensitive to mental workload across different tasks (Figure 6B). According to this theory, limited capacity is the fundamental characteristic of human cognitive resources (Wickens, 2008). That means that the residual cognitive capacity available for additional tasks or perceptual stimuli will be less if you are engaged in a more demanding task. The magnitude of recruited mental resources can be revealed by ERPs’ amplitudes and latencies (Ghani et al., 2020). Thus, high-load mental processing may use more attentional resources and reduce the brain’s capacity to recognize visual or auditory events (Wickens, 2008).

In search of an efficient workload classification method, Roy et al. (2016a) showed in a proof of concept that ERPs associated with passive auditory probes during the MATB achieved a classification accuracy above 80% for each participant, with minimal intrusiveness through the use of a single stimulus paradigm. However, Dehais et al. (2019) found that the classification accuracy using both ERPs and frequency-based features simultaneously did not surpass chance level in discriminating between high and low mental workload during a real flight scenario with a passive auditory Oddball task. The main results of this study revealed that low workload compared to high workload resulted in a higher P300 amplitude for the auditory target (Pz, P4 and Oz electrodes) along with higher alpha band power (Pz electrode) (Dehais et al., 2019). In another study, EEG/ERP measurements related to piloting tasks with 2 levels of mental workload showed that increased mental workload was accompanied by a lower P3b amplitude (Causse et al., 2015) (Figure 6B). A reduced P3b amplitude reflects the depletion of cognitive resources allocated to processing instructions (Causse et al., 2015). Consequently, these studies pave the way for the efficient use of ERPs for monitoring mental states in near-real-life environments, and contribute to the development of adaptive user interfaces (Roy et al., 2016b; 2016a).

In support of the theory of mental resources, some studies have shown that the amplitude of various ERPs components decreased when sensory stimuli were presented concurrently with the performance of other tasks (Jaquess et al., 2017). Scheer et al. (2016) discovered that steering demands on mental resources reduced the amplitudes of the early P3, late P3, and re-orientation negativity (RON) also call contingent negative variation (CNV) components of the ERPs elicited by task-irrelevant environmental sounds. The results observed in MATB tasks have shown that the amplitudes of tir-a (task-irrelevant auditory) ERPs components, including N1, early and late P3a, and the RON (CNV), decreased significantly with the increase in mental workload induced by two levels of difficulty, as they do for the N-back test (Ke et al., 2021). Additionally, the task type did not appear to have a significant influence on the amplitudes and topological layout of the mental workload-sensitive to tir-aERPs features (Ke et al., 2021). These authors suggested that the tir-aERPs potentially serve as more consistent indicators of mental workload across different task types compared to power spectral density (Ke et al., 2021) and should be considered in future task-independent mental workload monitoring studies.

Finally, the use of functional magnetic resonance imaging (fMRI) showed that static and dynamic functional connectivity between the default mode and dorsal attention networks was stronger during multi-than single-MATB tasking (Lam et al., 2022). In this study, further analysis showed that the connectivity profile of multitasking can use positive and negative connections to orchestrate various cognitive functions so that behavior remains accurate, efficient and dynamic, despite changes in workload.

## 4 MATB and environmental constraints

The MATB was used to study mental workload in interaction with environmental constraints (hypoxia/hyperoxia, physical exercise, sleep deprivation, heat/cold exposure, noise). In such cases, the difficulty of the MATB must be adapted to the physiological stress experienced by the person. However, little is known about this interaction, probably due to the limited availability of specific experimental tools or platforms (gas exposure, hypobaric or hyperbaric chamber, sleep lab). These studies are relevant to athletes, pilots and military personnel in operational conditions, where managing a high mental workload is crucial. Several articles, aimed at studying the impact of physiological constraints on mental workload, through MATB performance, were excluded from the systematic review because they included only one level of mental workload. In this part of the review, we have analyzed these articles in order assess the potential interest of MATB in this context and to improve the methodological recommendations.

### 4.1 The hypoxia environment

Exposure to a hypoxic environment (simulated by gas mixtures, or in a hypoxic chamber or high-altitude environment) has a deleterious effect on physiological and mental functions. Van Dorp et al. (2007) studied the effect of added inspired CO_2_ during artificially induced normobaric hypoxia (oxygen saturation approximately 80%, PETO_2_ = 40 mmHg) on MATB performance. During minutes “25–55” of hypoxia, brain oxygenation levels, measured by near-infrared spectroscopy, were significantly higher during CO_2_ inspiration (PETCO_2_ = 0.4 mmHg) than during no CO_2_ inspiration. Performance test results indicated a negative effect of hypoxia alone on the MATB tracking test (Van Dorp et al., 2007). These results demonstrate the ability of added inspired CO_2_ to improve performance during hypoxia by preventing the vasoconstriction of cerebral blood vessels induced by hypoxia-hypocapnia (Van Dorp et al., 2007).

In another study (included in the 19 selected studies) conducted in an enclosed environmental chamber, Zhang et al. (2020) exposed participants to elevated carbon dioxide (CO_2_) concentrations during MATB tasks with low, medium, and high mental workloads. The results showed that subjective mental workload (NASA-TLX scores) was not associated with CO_2_ concentration but was positively related to the different designed mental workloads. The overall MATB task performance (expressed as weighted response time calculated as the average ratio of response time to accuracy) decreased significantly as CO_2_ concentration increased from 1500 ppm to 3500 ppm, then returned close to 1500 ppm after 5000 ppm, with no difference between 3500 ppm and 5000 ppm or 1500 ppm and 5000 ppm (Zhang et al., 2020). In this study, event rates were lower than those reported in literature, with 9 events per minute (108 events for 12 min) for the high mental workload situation.

Recently, Bottenheft et al. (2023) investigated how cognitive performance, assessed using two multitasks, the MATB-II and the SYNWIN, is affected by the combination of two stressors relevant to pilots in military operations: heat load (induced by increasing ambient temperature to ∼28°C) and 45 min of hypobaric hypoxia (induced in a hypobaric chamber at a simulated altitude of 13,000 ft). The results showed that heat load was the main cause of reduced cognitive performance in MATB-II subjects (Bottenheft et al., 2023). Only a arithmetic sub-task of the SYNWIN test was sensitive to hypobaric hypoxia (Bottenheft et al., 2023). In another study, a 6-h exposure to 8,000 ft or 10,000 ft does not affect MATB performance (Bouak et al., 2019). Thus, moderate hypoxia does not appear to decrease MATB performance. A recent study confirmed that moderate hypoxia exposure (14.0% O_2,_ ∼3000 m) has a low effect on MATB-II performance, but induces a higher autonomic nervous system response during simultaneous exposure to high cognitive load (MATB) (Temme et al., 2023).

### 4.2 The diving environment

The potential effects of submarine and diving operations on mental workload and cognition must be considered (Sharma et al., 2023). Clearly, cognitive functions such as alertness, sensing, reaction time, perception, memory, learning, thinking and decision-making are crucial for diving safety. Furthermore, the most frequently observed acute effect of diving is gas narcosis, which results from the complex interaction of gases, activities, and environmental conditions. MATB-II simultaneously tests multiple demanding cognitive tasks involving motor performance, attention, impulsivity, memory and planning, all of which are also present in the realistic diving environment.

This is how Freiberger et al. (2016) exposed subjects to varied inspired partial pressures of CO_2_, N_2_, and O_2_ in immersed, exercising subjects while assessing multitasking performance with the MATB. Cognitive performance was tested under 20 conditions of gas partial pressure and exercise in 42 male subjects meeting U.S. Navy age and fitness profiles. They observed impairment of memory, attention, and planning, but not motor tasks, during N_2_ partial pressures of 4.5 ATA, partially rescued after exposure to sea-level O_2_. However, during hyperbaric situations, they observed decreased performance. This work helps to understand the relative contributions of factors associated with diving narcosis and toxicity, and could be useful in predicting the effects of gas mixtures and exercise conditions on the cognitive performance of divers.

### 4.3 The physical exercise constraint

Most of the work that has attempted to devise objective methods for modeling mental workload has been primarily based on neurological or physiological data collected when participants are not exercising. While these models may be useful for scenarios involving static operators, they can also be applied to real-life situations where operators perform multitasking at varying levels of physical activity (Albuquerque et al., 2020; Rosanne et al., 2021), such as those faced by first responders, firefighters and police officers.

Thus, several conventional EEG enhancement algorithms have been evaluated for their potential benefits in mental workload measurement in ecological contexts (Rosanne et al., 2021). This study showed that overall performance levels for mental workload measurement remained below those typically reported for stationary users, and the authors suggested that existing enhancement algorithms have been developed and optimized to remove muscle and blink/movement artifacts, and not necessarily the motion artifacts seen, for example, when running. In their next study, the authors proposed the use of an adaptive filter to remove movement-specific motion artifacts from mobile EEG data, and tested the algorithm on a database collected from 48 participants performing the MATB under two workload conditions (low and high) and two types of physical activity (stationary bike, 70 rpm and treadmill, 5 km/h), each at three activity levels (none, medium, and high). Their results show that the proposed algorithm accurately removes body movement artifacts and resulting in mental workload monitoring performance as high as 97%, independent of activity type and level (Rosanne et al., 2021).

Using the experimental protocol described above, the same research group made available the protocol of a multimodal database of mental Workload Assessment Under physical aCtivity (WAUC) including the raw data and features from six neural and physiological modalities: EEG, ECG, breathing rate, skin temperature, galvanic skin response (GSR), blood volume pulse (BVP), subjective ratings, and scripts to reproduce the experiments (Albuquerque et al., 2020).

### 4.4 The sleep loss/deprivation constraint

It is widely recognized that prolonged wakefulness leads to deterioration in work performance (Alhola and Polo-Kantola, 2007). One of the parameters most commonly used to assess the effect of sleep loss or deprivation is reaction time (RT) on the psychomotor vigilance task (PVT) (Basner and Dinges, 2011). However, the simplicity of the PVT is also its weakness, as it is thought to fail to assess other factors, such as working memory and multitasking performance. The MATB has been used to assess the human performance of aircrews during prolonged wakefulness (Kong et al., 2022). Although MATB offers greater complexity and varied measures compared to the PVT, it requires careful configuration of task difficulty levels to avoid learning effects. Furthermore, while MATB has shown promise in detecting performance degradation on complex tasks due to prolonged wakefulness, several studies have produced conflicting or unclear results regarding the number and type of MATB tasks to be used for this assessment (Lopez et al., 2012; Gutzwiller et al., 2014; Kong et al., 2022). For example, tracking RMSE was the only MATB measure to show significant changes (degradation) during prolonged wakefulness (Lopez et al., 2012). Indeed, Lopez et al. (2012) examined the effects of simulated flight and completed the MATB along with two other cognitive tests (PVT and OSPAN, Operation Span Task) at 3-h intervals over a 35-h sleep deprivation period, The MATB tracking Root Mean Square Error (RMSE) performance began declining earlier than other cognitive measures used, and these decrements lasted for the entire study, recovering slightly during the last session but never reaching baseline levels (Lopez et al., 2012). During the second half of the sleep deprivation period, simulated flight performance was well predicted by PVT, but much less so by the MATB.

Kong et al. (2022) analyzed pilot performance deterioration during 25 h of prolonged wakefulness, using both the MATB set at a high level for completion of the three tasks simultaneously (excluding the Tracking task), and PVT. They observed that all MATB tasks showed significant performance degradation during prolonged wakefulness. The MATB performance deteriorations were highly correlated with those observed from PVT measures, especially when the task difficulty level was high (i.e., simultaneous tasks) compared to those of a lower difficulty (i.e., single SYSMON and COMM tasks). The authors concluded that MATB is an effective tool for analyzing performance deterioration during a prolonged period of wakefulness, similar to PVT, but with the added benefit of providing more realistic aviation cockpit simulation scenarios.

Thus, with appropriate levels of difficulty, MATB can be used as a suitable simulation tool to study the effects of prolonged wakefulness on aircraft pilots (Kong et al., 2022).

## 5 Discussion

In aviation, complex socio-technical environments such as airliners, combat aircraft, and non-autonomous UAVs (unmanned aerial vehicle) are examples where human multitasking performance is evident. In these systems, the pilot constantly monitors the information generated in different subsystems, interprets it simultaneously using his processing sources, and makes the appropriate decision based on the situational awareness he or she has acquired.

The NASA MATB has been shown to predict prospective memory performance during complex simulated flight (van Benthem et al., 2019). Prospective memory is strongly related to executive functions such as multitasking and cue detection (Dismukes and Nowinski, 2007). Van Benthem et al. (2019) reported the relationship between the MATB and prospective memory during a simulated flight with fifty-one pilots. The results showed that the pilot’s level and number of flying hours were not correlated with any of the MATB subtests, but recent pilot-in-command hours were negatively correlated with SYSMON errors in the medium and high difficulty levels. In addition, the number of years licensed was positively correlated with SYSMON errors, although authors explained that this is most likely an artifact of the negative effect of age on performance (van Benthem et al., 2019). The MATB has thus been used in studies of civilian (Kong et al., 2022) and military pilots F-117 A and helicopter (Caldwell et al., 2004; Bouak et al., 2019) and has been considered relevant to assess pilot workload and performance deterioration during prolonged wakefulness, diving or hypoxia exposure.

The increase in MATB-induced mental workload has been validated in comparison to decreased behavioral MATB performance (Hancock et al., 1995) and increased subjective workload scores (Hancock et al., 1995; Chandra et al., 2015). This increase is also associated with changes in different electrophysiological indices of mental workload (Chandra et al., 2015; Ke et al., 2015) and fMRI responses (Lam et al., 2022). The latter study revealed that the connectivity strength between large-scale cognitive networks, specifically the DMN-DAN (default mode network-dorsal attention network) and DAN-FPN (frontoparietal network), differed when participants were multitasking compared to single tasking. Another advantage of choosing MATB over others is its ability to provide objective scoring of mental workload at different load levels, similar to the NASA-TLX questionnaire, which has been validated by an extensive laboratory research program (Hart and Staveland, 1988).

A recent exploratory study conducted by the Naval Submarine Medical Research Laboratory investigated the ability of a 30 min 10-task cognitive test battery, covering a range of cognitive functions, to predict performance in the MATB complex task (Peltier et al., 2022). This study aimed to identify individuals who could sustain visual and auditory attention, task switch, and react quickly and accurately. This battery was chosen because it includes measures of several cognitive abilities that have demonstrated success in predicting performance in unrelated outcome tasks. It includes testing of sensorimotor speed, memory for complex figures, working memory capacity, executive functioning, spatial orientation, the ability to recognize emotions conveyed through facial expressions, abstract reasoning and pattern recognition, complex screening and tracking, risk-taking behavior, and vigilance. Results showed that MATB, performance significantly declined over time, confirming subjects’ vigilance decrement. A regression using performance on the cognitive battery found that such performance accounted for 51% of the variance in overall MATB performance and 31% of the variance in sustained attention. However, the literature as a whole suggests that it is very difficult to extrapolate the specific cognitive functions altered by the MATB task.

While MATB has shown promise for detecting performance degradation due to high workload, fatigue, prolonged wakefulness or physiological constraints, studies have produced contradictory or unclear outcomes regarding MATB configurations. The main limitations for the use of the MATB simulator include the relative low number of publications, only 19 in our work, the lack of description of the MATB task design and large number of differences in the design of the simulator complexity. While the median duration seems not to be a factor of mental workload, the complexity induced by event rates, the number of tasks used and the overlap are workload factors that may be included in the software design. However, the event rates used in publications vary widely, with low workload levels (median: 3 events/min, range: 0.6–17.2), and high workload levels (23.5 events/min, range: 9–65). The common factor between the studies is probably a 6-fold increase between low and high mental workload for the event rate.

## 6 Recommendations

The challenge for the current use of the MATB simulator is to increase reproducibility and comparability between studies. To this end, we provide recommendations for standardizing MATB descriptions in literature and study design (Table 5). This is a challenge in the context of increased human-machine interactions and digital influx in the future for pilots. Moreover, MATB seems to be pertinent to study mental workload in interaction with different environmental/operational constraints. Research on the effect of environmental/operational constraints combined with mental workload is a future challenge in the fields of space, aeronautics, military and sports.

**TABLE 5:** Recommendations for the conception of MATB script file and description in publications.

Aim	Methods
Limit the learning effect	Practice a training, at least 3 × 5 min, with increased workload levels
Create a multitasking configuration	Use the 4 tasks simultaneously
Limit a potential order effect bias	Randomize levels of mental workload
Limit the inverted U-shape effect on performance	Use more than 2 mental levels configuration
Create a multitasking task	Ask subjects to perform all 4 tasks, without task prioritization
Assess subjective workload	Use at least the NASA-TLX (To improve comparability between studies)
Assess MATB performance	Use identical manual tracking configuration and calculate RMSE at all levels (to improve comparability between studies)
Describe subjects’ characteristics	Number, age, piloting expertise (To improve comparability between studies)
Describe the variables and methods used to objectify the increase in mental workload	Give results (mean and variability) of physiological and neurophysiological parameters
Describe precisely the MATB design	Provide a table in the method, with for each level: - duration of the level, - number of stimuli for each task (event rate), - ratio of target/distractor of each task, - inter stimuli periods (overlap)

Nevertheless, when MATB is associated with environmental/operational constraints such as sleep deprivation (Caldwell et al., 2004; Kong et al., 2022), hypoxia/altitude (Van Dorp et al., 2007; Bottenheft et al., 2023) or high levels of carbon dioxide (Zhang et al., 2020), high mental workload level is induced by lower events rates. There is also great interest in this situation to assess the interaction between environmental operational constraints and mental workload, with at least two levels of mental workload and two physiological situations (Zhang et al., 2020).

In order to limit the effect of learning on performance (Miyake et al., 2009; Kim et al., 2019), we recommend a training session to become familiar with the MATB tasks and observe stable performance. Training is characterized by high interindividual variability, but short, repeated 5-min training sessions seem sufficient (Kim et al., 2019).

Doing all the subtasks is necessary to create a multitasking MATB configuration, which is the main objective of this test. High mental workload could be created using one or two subtasks, but these configuration create specific constraints (Oswald et al., 2007; Lam et al., 2022). Only, multitasking MATB configuration has been validated in comparison to simulated flight (Caldwell, 1999; Caldwell et al., 2004; Bouak et al., 2019).

In order to limit the impact of increasing level (Bowers et al., 2014). and transition effects, we recommend to randomize mental workload levels. The main methodological difficulty of MATB is to observe a performance gap between the two levels, induced by a small difference in mental workload or by the effect of inverted U-shape on performance (Yerkes et al., 1908; Arent and Landers, 2003). We therefore recommend testing the increase in mental load using changes in an objective parameter (such as tracking performance) and a subjective scale (such as NASA-TLX). The use of these two parameters will improve comparability between studies. Finally, evaluation of certain psychophysiological and neurophysiological markers that reliably characterize mental workload levels could be among the recommendations (Miyake et al., 2009; Bowers et al., 2014; Charles and Nixon, 2019).

In some studies, optimal performance was observed with a moderate event rate (Zhang et al., 2020). This methodological point is fundamental for the design of the MATB to observe a significant decrease in performance and a significant difference between the MATB levels. This could be achieved by using an additional level of moderate workload as in Zhang’s study (Zhang et al., 2020). In the same way, in order to improve reproducibility between studies and meta-analyses, we recommend to improve the description of the different MATB levels (i.e. duration of the level, number of stimuli for each task (event rate), ratio of target/distractor of each task and inter stimuli periods), as well as main subjects’ characteristics (number, age, piloting expertise … ) (Benthem et al., 2019).

## 7 Conclusion

Real-time monitoring of mental workload is a crucial step in building closed-loop adaptive support systems for human-machine systems. Due to recent technological developments and the continuing growth in automation levels, humans in operational environments are expected to work with more complex systems, where multitasking performance becomes an important issue. The MATB could be used to assess workload at different levels using multitasking configurations. Although MATB has shown promise for detecting performance degradation due to high workload, fatigue, prolonged wakefulness or physiological stresses, studies present conflicting or unclear results regarding MATB configurations. We propose recommendations for standardizing MATB design, configuration description and training to enhance reproducibility and comparison between studies. This poses a challenge in the context of increasing human-machine interaction and digital influx in the future for pilots.
